# Unexpectedly long incubation period of *Plasmodium vivax *malaria, in the absence of chemoprophylaxis, in patients diagnosed outside the transmission area in Brazil

**DOI:** 10.1186/1475-2875-10-122

**Published:** 2011-05-14

**Authors:** Patrícia Brasil, Anielle de Pina Costa, Renata Saraiva Pedro, Clarisse da Silveira Bressan, Sidnei da Silva, Pedro Luiz Tauil, Cláudio Tadeu Daniel-Ribeiro

**Affiliations:** 1 Instituto de Pesquisa Clínica Evandro Chagas (IPEC), Fundação Oswaldo Cruz (Fiocruz), Rio de Janeiro. Av. Brasil 4365. Manguinhos, Rio de Janeiro, RJ - CEP 21.045-900, RJ, Brazil; 2 Centro de Pesquisa Diagnóstico e Treinamento em Malária (CPD-Mal), Fiocruz and Secretaria de Vigilância em Saúde (SVS) - Ministério da Saúde (MS), Brazil; 3 Núcleo de Medicina Tropical. Área de Medicina Social, Faculdade de Medicina, Universidade de Brasília, Brasília - CEP 70.910-900, Brazil; 4 Laboratório de Pesquisas em Malária. Instituto Oswaldo Cruz, Fiocruz. Pavilhão Leônidas Deane - 5° andar. Av. Brasil 4365. Manguinhos, Rio de Janeiro, RJ - CEP 21.045-900, RJ, Brazil

## Abstract

**Background:**

In 2010, Brazil recorded 3343,599 cases of malaria, with 99.6% of them concentrated in the Amazon region. *Plasmodium vivax *accounts for 86% of the cases circulating in the country. The extra-Amazonian region, where transmission does not occur, recorded about 566 cases imported from the Amazonian area in Brazil and South America, from Central America, Asia and African countries. Prolonged incubation periods have been described for *P. vivax *malaria in temperate climates. The diversity in essential biological characteristics is traditionally considered as one possible explanation to the emergence of relapse in malaria and to the differences in the duration of the incubation period, which can also be explained by the use of chemoprophylaxis. Studying the reported cases of *P. vivax *malaria in Rio de Janeiro, where there is no vector transmission, has made it possible to evaluate the extension of the incubation period and to notice that it may be extended in some cases.

**Methods:**

Descriptive study of every malaria patients who visited the clinic in the last five years. The mean, standard deviation, median, minimum and maximum of all incubation periods were analysed.

**Results:**

From the total of 80 patients seen in the clinic during the study time, with confirmed diagnosis of malaria, 49 (63%) were infected with *P. vivax*. Between those, seven had an estimated incubation period varying from three to 12 months and were returned travellers from Brazilian Amazonian states (6) and Indonesia (1). None of them had taken malarial chemoprophylaxis.

**Conclusions:**

The authors emphasize that considering malaria as a possible cause of febrile syndrome should be a post-travel routine, independent of the time elapsed after exposure in the transmission area, even in the absence of malaria chemoprophylaxis. They speculate that, since there is no current and detailed information about the biological cycle of human malaria plasmodia's in Brazil, it is possible that new strains are circulating in endemic regions or a change in cycle of preexisting strains is occurring. Considering that a prolonged incubation period may confer advantages on the survival of the parasite, difficulties in malaria control might arise.

## Background

The malaria incubation period is defined as the time elapsed between exposure to the infectious agent (through the bite of the *Anopheles *mosquito) and the manifestation of the first clinical sign or symptom. Usually, these periods vary depending on the species of *Plasmodium *causing malaria. The average incubation period is 9-14 days for *Plasmodium falciparum*, 12-17 days for infections by *Plasmodium vivax *and 18-40 days for infections caused by *Plasmodium malariae *[1].

The relapse patterns and variations in the length of the incubation period, including a delay of four months or longer, was first described by Korteweg in Holland between 1901 and 1902 (cited by Swellengrebel and De Buck [2]). Later, in 1935, Nikolaev proposed that there were two strains of *P. vivax *(cited by Tiburskaya [3]) with different incubation periods and gave the sub-specific taxonomic name of *P. vivax hibernans *to the variety with the longest incubation period. It was suggested that this sub-species had adapted to more northern latitudes where the anopheles vector was absent for much of the year. Shute (1946) [4] proposed that the sporozoite infective inoculum would be inversely related to the prepatent and incubation period. However, in Moscow, Tiburskaya [3] demonstrated situations in which the length of the incubation period did not depend on the number of inoculated sporozoites, but instead was determined by the inherent properties of the strains. It was also believed that strains with prolonged latency could be attributed either to the "senility" of the sporozoite towards the end of the season or to the low number of sporozoites in the infective bite [5].

According to Shute [5], the differences between the *P. vivax *strains could be explained by the assumption that, in varying proportions, all strains of *P. vivax *produce two types of sporozoites: one eliciting short prepatent periods (Type I) and the other lying dormant or developing slowly to give rise to long prepatent periods (Type II). In this model, the latter type would greatly predominate in "temperate strains", but not in tropical ones. It was thought that relapses of *P. vivax *could in reality correspond to a delayed parasitaemia arising from Type II sporozoites. In the same year, Garnham stated that the length of the incubation period was considered the major biological difference between Dutch, Madagascar, and USSR strains, and although there was no evidence of specifically dormant forms, it was believed that if certain sporozoites failed to develop in the normal time, they could be reactivated by an unknown factor one year or more after inoculation [6].

In 1980, Warwick [7] proposed that the ambient winter temperatures could extend the incubation period of *P. vivax *in humans, based on the concept that temperatures persistently above a minimum of 23.9°C were required for sporozoite maturation [8], thereby limiting vector transmission in cold areas. Finally, in 2007, Nishiura *et al *in Korea [9] suggested that the incubation periods would likely reflect adaptation to the behaviour of the principal vector of the region, which hibernates during the winter season. Currently, several reports associate the extension of the incubation period to malaria prophylaxis among travellers [10,11].

The opportunity to study some cases of *P. vivax *malaria in Rio de Janeiro, where there is no vector transmission, has made it possible to detect and to evaluate certain peculiar aspects of the natural evolution of the disease in human beings. One main aspect was the extension of time required for the parasites to progress through liver schizogony and produce symptoms by their propagation in the bloodstream.

*Plasmodium vivax *infections with prolonged periods of incubation and no association with malaria prophylaxis in patients from the Amazon region in Brazil and in one patient from Indonesia are presented.

In addition to demonstrating an interesting clinical situation and the need for clinicians to consider the diagnosis of malaria in a patient presenting symptoms a long time after exposure, even in the absence of chemoprophylaxis, our cases raise questions regarding the understanding of the biology of the host/*P. vivax *interactions.

## Methods

### Design and study location

This is a descriptive study conducted at the Acute Febrile Disease Outpatient Clinics of IPEC, Fiocruz, a specialized post-travel care clinic located in Rio de Janeiro, from January, 2005, to February, 2010.

### Selection of patients

All malaria patients presenting clinical signs or symptoms of malaria and positive thick blood smears were enrolled in the study. The following variables of interest were recorded: estimated incubation period, place and year of infection, date of diagnosis, previous malaria history and year of the first malarial infection. No patients had malaria prophylaxis, had not received blood transfusions nor had close contact with a person with malaria after departure from the endemic area. No patients had haemoglobinopathies. Because it was not possible to determine the date of exposure to the infective mosquito bites, the minimum incubation period was estimated based on literature (9 days for *P. falciparum *and 12 days for *P. vivax*) [1]. The maximum was estimated by the interval between the day of the return from the malaria transmission area until the first day of symptoms. The mean, standard deviation, median, minimum and maximum of all incubation periods are shown in Table 1. Each patient gave fully informed consent. Children were not included.

**Table 1:** Time between the day of the return from the malaria transmission area and the first day of onset of symptoms of malaria cases diagnosed in the Acute Febrile Diseases Clinic, Rio de Janeiro (2005 until January 2010)

	*P. vivax*	*P. falciparum*	Mixed Infection *(P. vivax *and *P. falciparum)*
Mean	45 d	10 d	11 d

Standard Deviation	67 d	16 d	7 d

Median	25 d	6 d	12 d

Maximum	360 d	60 d	18 d

Minimum	12 h	12 h	12 d

d = days; h = hours

The project was submitted and approved by the Ethical Committee in Research of the Instituto de Pesquisa Clínica Evandro Chagas (IPEC), Fiocruz (number 0020.0.009.000-07), maintaining strict secrecy and confidentiality of the information obtained.

### Detection and quantification of malaria parasites

Thin and thick blood smears were stained with Giemsa and analysed by light microscopy using an immersion oil lens (X100 objective magnification) to identify the parasite species and determine the density of *Plasmodium *asexual and sexual stages, according to standard procedures [12]. Each smear was evaluated separately by two expert microscopists who had been blinded to the clinical status of the patients.

### Data analysis

All information was recorded on a standardised form for study and subsequently entered into a database using Statistical Package for Social Sciences (SPSS). SPSS-WIN 16.0 was also used for data analysis.

## Results

During the study period, 80 malarious patients were diagnosed and treated. Of them, 50 (62.5%) presented with *P. vivax *malaria, 20 (25%) with *P. falciparum *malaria, eight (10%) with mixed (*P. vivax*/*P. falciparum*) infection and two (2.5%) with *P. malariae*. All patients were travellers, most of them (51) from the Amazon region, in Brazil; 17 travelled from Africa, 11 were from South and Central America, and one was from Indonesia.

Time between the day of return from the malaria transmission area and the first day of onset of symptoms recorded for all patients diagnosed in the Acute Febrile Diseases Clinic Rio de Janeiro (2005 until January 2010) was four times longer for *P.vivax *than for *Plasmodium falciparum *and is illustrated in Table 1. The estimated mean incubation period for all cases was 31 days (SD 51 days), with a median of 12 days and extreme values of 9 and 360 days.

**Figure 1:**
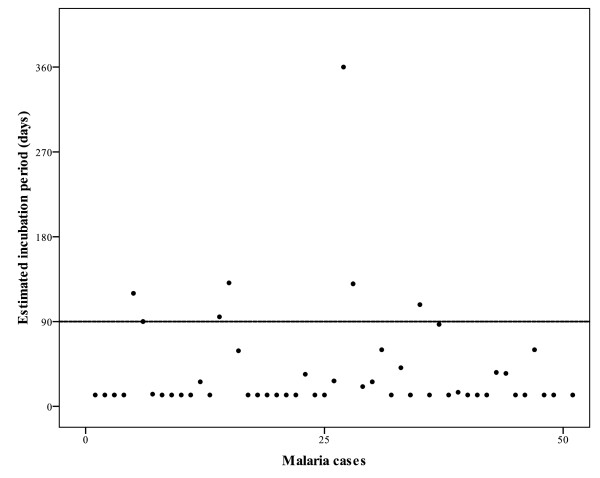
**Estimated incubation period in days for each *P. vivax *infected patient**.

An estimated incubation period longer than 90 days was observed in seven (14%) of the patients with *P. vivax *malaria (Figure 1). The average incubation period (147 days) among this group was about twelve times longer than the classical period described in the literature (12 days). Malaria was contracted during visits to the Amazonian region (in six cases) and Indonesia (in one). Their details are described in Table 2. There were no differences in clinical presentation between individuals with *P. vivax *infection with different incubation periods. No patient had undergone malaria chemoprophylaxis or had taken any pharmacological drug that could inhibit the parasite's development.

**Table 2:** Patients with *P. vivax *infection and estimated incubation period ≥ 90 days

	Departure from endemic area	Date of initial symptoms (days after departure)	Possible local of infection	Previous malaria	Year of previous infection	Relapse
**Patient 1**	06/30/2007	07/31/2008 (360)	Indonesia	Yes	2007	Yes
**Patient 2**	12/12/2005	04/23/2006 (131)	Rondonia State, Brazil	Yes	2001	Unknown
**Patient 3**	12/3/2007	04/12/2008 (130)	Amazonas State, Brazil	No	NA	No
**Patient 4**	02/9/2005	06/16/2006 (120)	Roraima State, Brazil	Yes	2000	Unknown
**Patient 5**	12/6/2007	03/24/2008 (108)	Amapa State, Brazil	Yes	2007	Yes
**Patient 6**	07/5/2006	10/9/2006 (95)	Para State, Brazil	Yes	2006	Yes
**Patient 7**	06/15/2005	09/15/2005 (90)	Para State, Brazil	Not Avaiable	Not Avaiable	Unknown

NA = non aplicable

## Discussion

This is the second report of prolonged incubation period of malaria in patients without chemoprophylaxis coming from an endemic area in Brazil. A recent paper by one of the authors of this report (Tauil PL) described three cases of vivax malaria originating from the Amazon region and diagnosed in Brasilia, Federal District, six months after departure from the endemic region in 2008 [13]. Two of those patients were infected in the same town (São Gabriel da Cachoeira, Amazonas State, Brazil), as one of the patients in the present study. Some of the cases in this study were detected in 2005 and 2006, prior to the cases detected in Brasilia and reported by Tauil *et al *[13]. All possible current explanations for these prolonged periods (use of malaria prophylaxis or other pharmacological drugs that would inhibit the *Plasmodium *development; blood transfusions; close contact with a person with malaria after departure from the endemic area or haemoglobinopathies) were eliminated. The observation of a longer incubation period (≥90 days) in 14% of the *P. vivax *malaria patients seen at IPEC, in Rio de Janeiro, may indicate the importance of monitoring these characteristics worldwide, as it may represent an evolutionary change in *P. vivax *behaviour. The average incubation period of *P. vivax *malaria presented here was approximately twelve times longer than the classical period described in the literature. In this study, the extended incubation time occurred in both prime-infected (130 days) and non-prime-infected (131 days) patients, so the possibility of relapse among non-prime-infected patients cannot be ruled out. However, in two patients previously infected with malaria, the period between the last infection and the current clinical manifestation was five and six years, by far exceeding the maximum period of relapse reported for *P. vivax *(three years) [14]. Cities such as Rio de Janeiro, as well as areas in the northern hemisphere without disease transmission may be considered strategic places for monitoring incubation period, clinical cures and treatment failure in cases of malaria, facilitating the identification of the above features without misinterpreting variations as the result of new infections.

During the five years of surveillance (2005-2010) no seasonal differences in the prevalence of clinical *P. vivax *malaria diagnosed outside the endemic area were observed between these cases with prolonged incubation periods. Regardless, the postulate that extended incubation periods may represent an adaptation of the species to overcome cold temperatures, thereby conferring advantages for the survival of the parasite, does not seem to fit the reality of tropical areas, where the temperature is rarely below 10°C. Although the role of strain-specific variation in prolonged incubation periods has been questioned by some authors [9], it is possible that new strains of *Plasmodium *are circulating in tropical areas, especially in the Amazon, which is a region frequently visited by foreigners and which has seen the movements of troops.

Fever is one of the most common clinical signs in returning travellers [15-20]. The incubation periods of potential pathogens should be considered when formulating differential diagnoses. The geographic location(s) visited, the traveller's activities and the frequency of specific diseases in the region are usually taken into account. According to the observations reported here, malaria should be considered among the diseases with longer incubation periods (weeks to months after return), even in patients without malaria chemoprophylaxis.

## Conclusions

It is classically considered that the co-existence of short and long-term incubation periods may imply that prolongation of this phase is either a genetically regulated feature of parasites or is controlled within *Anopheles *spp. by mechanisms yet to be defined. Therefore, new molecular tools need to be used for investigation of biological characteristics and origin of the *Plasmodium *strains that presents a prolonged incubation time in Brazilian patients that have never visited the temperate zone.

*Plasmodium vivax*, responsible for 86% of malaria cases in Brazil [21], has long been neglected and mistakenly [22]. The change in incubation period reported here is particularly important in theory, because it raises the possibility of changes in the biology and evolution of this organism, entering into strategic debates taking place on malaria epidemiology and control; and in practice because malaria is one of the most important infectious diseases among travellers and a long incubation period is one of the causes of missing early malaria diagnosis.

## Competing interests

The authors declare that they have no competing interests.

## Authors' contributions

PB - responsible for conception and design of the work, interpretation of data and drafting the manuscript.

APC - analyzed data, made the literature review and helped drafting the manuscript.

RSP - helped analyzing the data and reviewed the text.

CSB - responsible for the production of data and helped reviewing the text.

SS - carried out the parasitological examinations and helped in the literature review.

PLT - helped in interpretation of data, literature review and reviewing the manuscript.

CTDR - helped in the design of the work and reviewed the text up to the final version to be published.

All authors read and approved the final manuscript.

## Financial support

This work was supported by CGLAB from the Secretaria de Vigilância em Saúde to the Centro de Pesquisa Diagnóstico e Treinamento em Malária (CPD-Mal), Fiocruz, Ministério da Saúde, Brazil.
